# Opposing Changes in Synaptic and Extrasynaptic N-Methyl-D-Aspartate Receptor Function in Response to Acute and Chronic Restraint Stress

**DOI:** 10.3389/fnmol.2021.716675

**Published:** 2021-10-08

**Authors:** Yiu Chung Tse, Moushumi Nath, Amanda Larosa, Tak Pan Wong

**Affiliations:** ^1^Neuroscience Division, Douglas Research Centre, Montreal, QC, Canada; ^2^Integrated Program in Neuroscience, McGill University, Montreal, QC, Canada; ^3^Department of Psychiatry, McGill University, Montreal, QC, Canada

**Keywords:** brain slice, corticosterone, electrophysiology, hippocampus, synaptic plasticity

## Abstract

A pertinent mechanism by which stress impacts learning and memory is through stress-induced plastic changes in glutamatergic transmission in the hippocampus. For instance, acute stress has been shown to alter the expression, binding, and function of the ionotropic glutamate N-methyl-D-aspartate receptor (NMDAR). However, the consequences of chronic stress, which could lead to various stress-related brain disorders, on NMDAR function remain unclear. While most studies on NMDARs focused on these receptors in synapses (synaptic NMDARs or sNMDARs), emerging findings have revealed functional roles of NMDARs outside synapses (extrasynaptic NMDARs or exNMDARs) that are distinct from those of sNMDARs. Using a restraint stress paradigm in adult rats, the objective of the current study is to examine whether sNMDARs and exNMDARs in the hippocampus are differentially regulated by acute and chronic stress. We examined sNMDAR and exNMDAR function in dorsal CA1 hippocampal neurons from brain slices of adult rats that were acutely (1 episode) or chronically (21 daily episodes) stressed by restraint (30 min). We found that acute stress increases sNMDAR but suppresses exNMDAR function. Surprisingly, we only observed a reduction in exNMDAR function after chronic stress. Taken together, our findings suggest that sNMDARs and exNMDARs may be differentially regulated by acute and chronic stress. Most importantly, the observed suppression in exNMDAR function by both acute and chronic stress implies crucial but overlooked roles of hippocampal exNMDARs in stress-related disorders.

## Introduction

N-methyl-D-aspartate receptor (NMDAR) is an ionotropic glutamate receptor that mediates synaptic plasticity and neuronal fate (Hardingham and Bading, 2003; Lau and Zukin, 2007; Paoletti et al., 2013). NMDARs also play crucial roles in mediating the biological impacts of stress on the nervous system. For instance, the facilitating effect of acute stress on pain sensation (Alexander et al., 2009) and fear conditioning (Shors and Servatius, 1995; Shors and Mathew, 1998) can be abolished by NMDAR antagonists. The atrophy and loss of spines on cortical and hippocampal neurons in stressed rodents can also be rescued by the blockade or the genetic knockdown of NMDARs (Magarinos and McEwen, 1995; Christian et al., 2011; Li N. et al., 2011; Martin and Wellman, 2011). The impact of stress on NMDAR expression and function has been extensively examined. Acute stress can increase the expression (Gore et al., 2000; Yamamoto et al., 2008; Yuen et al., 2012), binding (Krugers et al., 1993) and function of NMDARs (Yuen et al., 2009, 2011). The effect of chronic stress on NMDAR expression and function is less clear. Previous studies have revealed increased (Calabrese et al., 2012; Costa-Nunes et al., 2014; Pacheco et al., 2017), decreased (Pacheco et al., 2017) or stable (Schwendt and Jezova, 2000; Nasca et al., 2015) NMDAR expression in the hippocampus after chronic stress exposure. These somewhat conflicting findings may be due to differences in the type and duration of stressors used in these studies. Finally, only a few studies have examined the impact of chronic stress on NMDAR function (Karst and Joels, 2003; Yuen et al., 2012; Tse et al., 2019). Since chronic stress is associated with the pathogenesis of mood disorders (McEwen, 2003; Hammen, 2005), it is imperative to further examine if NMDAR function can also be regulated by chronic stress.

While our current understanding of the functional roles of NMDARs is based on studies of these receptors in synapses (sNMDARs), emerging findings have revealed functional roles for NMDARs that are located outside synapses (extrasynaptic NMDARs or exNMDARs). With high levels in the early postnatal age (Tovar and Westbrook, 1999), the proportion of exNMDAR decreases with development. However, close to 30% NMDARs in adult hippocampal slices remain extrasynaptic (Papouin et al., 2012). In addition to its substantial presence in the brain, exNMDARs can be physiologically activated through various means (for review, see Petralia et al., 2010; Papouin and Oliet, 2014). For instance, exNMDARs can be activated by glutamate from extracellular space (Le Meur et al., 2007), astrocytes (Carmignoto and Fellin, 2006; Nie et al., 2010) and repetitive stimulation of glutamate synapses (Suzuki et al., 2008). Back-propagating actional potentials also facilitate exNMDAR activation (Wu et al., 2012). Functional roles mediated by exNMDARs include neuronal synchrony (Fellin et al., 2004), synaptic computation (Oikonomou et al., 2012) and plasticity (Lu et al., 2001; Liu et al., 2013). These findings provide a more complete picture of exNMDAR physiology in addition to its contribution to neuronal death (Hardingham and Bading, 2010; Parsons and Raymond, 2014). Notably, these emerging functional roles of exNMDARs beg the question of whether these receptors are subjected to stress-related regulation.

Using a restraint stressor, the objective of this study is to examine the impact of acute and chronic stress on sNMDAR and exNMDAR functions in the hippocampal CA1 region, a region that is sensitive to stress and highly implicated in stress-related mood disorders (Brown et al., 1999; McEwen, 1999; Campbell and Macqueen, 2004; Duman and Monteggia, 2006). We found that exNMDAR function can be reduced by both acute and chronic stress.

## Materials and Methods

### Animals

All care and use of animals were in accordance with the guidelines and policies of the Canadian Council on Animal Care and approved by the Facility Animal Care Committee of the Douglas Research Centre (DRC), McGill University (animal use protocol number 2010-5935). Adult (8–12-week old) male Sprague Dawley (SD) rats were obtained from *Charles River Laboratories*. They were housed in the DRC animal facility and maintained on a 12/12 light cycle with lights on at 08:00 AM. Food and water were available *ad libitum*. Rats were used for experiments after being housed in the animal facility for at least 1 week to reduce the influence of stress from transportation.

### Stress Paradigm

Each episode of restraint stress was performed by putting a rat into a DecapiCone (*BrainTree Scientific*) for 30 min. For acute stress, rats were sacrificed 30 min after the end of one episode of restraint stress. For chronic stress, rats were restrained once daily for 21 days. Rats were sacrificed within a week after chronic stress (3.0 ± 0.4 days) for electrophysiological recording. Control rats for the acute stress and chronic stress experiments were handled daily for 1 and 21 days, respectively.

### Brain Slice Preparation

Unless specified otherwise, all materials and chemicals were purchased from *Sigma Aldrich*. Rats were anesthetized with isoflurane (5%) and decapitated using a guillotine. Trunk blood was collected after decapitation in EDTA-containing tubes to prepare serum for CORT (corticosterone) ELISA (*Abcam*. ELISA was performed using manufacturer’s procedures). Coronal brain slices were cut in a hyperosmotic, ice-cold and carbogenated (5% CO_2_, 95% O_2_) slice-cutting solution (in mM: 252 sucrose, 2.5 KCl, 4 MgCl_2_, 0.1 CaCl_2_, 1.25 KH_2_PO_4_, 26 NaHCO_3_ and 10 glucose, ∼360 mOsmol/L) using a vibrating blade microtome (*Leica*). Slices were then transferred to carbogenated artificial cerebrospinal fluid (aCSF; in mM: 125 NaCl, 2.5 KCl, 1 MgCl_2_, 2 CaCl_2_, 1.25 NaH_2_PO_4_, 26 NaHCO_3_ and 25 glucose, ∼310 mOsmol/L) at 32°C for 1 h, followed by room temperature incubation before recordings.

### Electrophysiology

#### General Procedures

All recordings were performed in the dorsal hippocampal CA1 region at room temperature. Synaptic responses were evoked by stimulating the Schaffer collateral-commissural pathway through a tungsten bipolar electrode (*FHC*) using constant current pulses (0.08 ms) at 0.05 Hz. Electrophysiological data were amplified by the Multiclamp 700 B amplifier (*Molecular Devices*), digitized by the Digidata 1440 (*Molecular Devices*), and stored in a PC for offline analysis.

#### Field Excitatory Postsynaptic Potential

Recordings of evoked AMPA receptor (AMPAR)-mediated field excitatory postsynaptic potentials (fEPSPs) were performed with an aCSF-containing glass pipette in the *stratum radiatum*. GABA_A_ receptors (GABA_A_Rs) were blocked by bicuculline methobromide (10 μM) and picrotoxin (20 μM). To record NMDAR-mediated-fEPSP, we reduced the concentration of MgCl_2_ in aCSF to 0.05 mM. AMPARs were blocked by DNQX (20 μM).

#### Excitatory Postsynaptic Current

**FIGURE 1:**
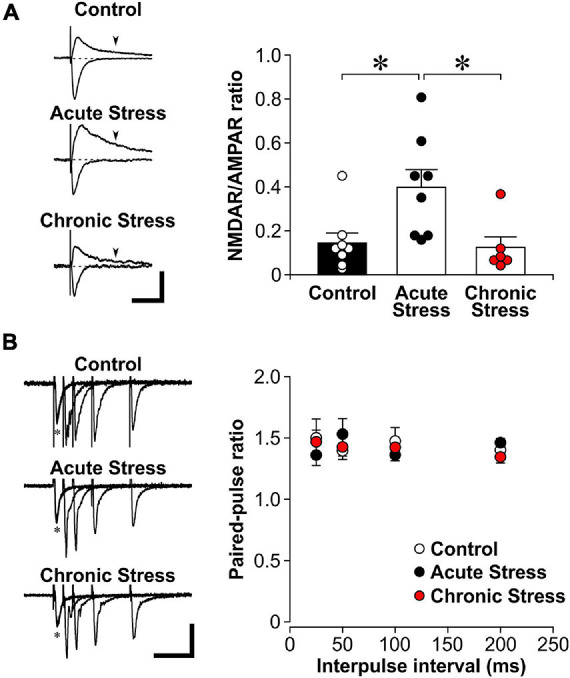
Acute stress enhances the NMDAR/AMPAR ratio. **(A)** *Left*: Representative traces of evoked excitatory postsynaptic currents (EPSCs) while voltage clamping hippocampal CA1 neurons at either –60 mV (inward currents) or +40 mV (outward currents). The AMPAR component is represented by the EPSC amplitude recorded at –60 mV. The NMDAR component is estimated by the magnitude of +40 current at 150 ms post stimulation (arrowheads), when the AMPAR component has returned to baseline (dotted line). Scale bar = 100 pA, 100 ms. *Right:* Histograms of NMDAR/AMPAR ratio in EPSCs recorded from control (8 recordings, 4 rats), acutely stressed rats (8 recordings, 4 rats) and chronically stressed rats (6 recordings, 3 rats). Mean ± SEM. *p < 0.05, *post hoc* Wilcoxon test after Kruskal-Wallis test. **(B)** *Left:* Representative traces of paired-pulse stimulation of evoked field excitatory postsynaptic potential (fEPSP) at different interpulse intervals (*represent the first pulse, followed by the second pulse at 25, 50, 100, or 200 ms in overlapping traces) that were recorded from brain slices of control, acutely stressed and chronically stressed rats. Scale bar = 0.2 mV, 100 ms. *Right:* Scatter plots show the paired-pulse ratios of fEPSP slope in control (12 recordings, 5 rats), acutely stressed rats (12 recordings, 5 rats) and chronically stressed rats (15 recordings, 4 rats). Mean ± SEM.

Recordings of evoked excitatory postsynaptic currents (EPSCs) from CA1 pyramidal neurons were performed using a patch pipette containing (in mM) 110 Cs-gluconate, 17.5 CsCl, 2 MgCl_2_, 0.5 EGTA, 10 HEPES (*Wisent Inc.*), 4 ATP, and 5 QX-314 (*Alomone Labs*), with the pH adjusted to 7.2 by CsOH (∼290 mOsmol/L). Only recordings with an access resistance lower than 20 MΩ were kept. We did not observe differences between the input resistance of CA1 neurons from different animal groups (control: 152.3 ± 6.4 MΩ; acute stress: 159.9 ± 7.0 MΩ; chronic stress: 162.1 ± 14.0 MΩ). The AMPAR- and NMDAR-mediated component of EPSCs were evoked while voltage clamping the membrane potential at –60 and + 40 mV, respectively. In EPSCs evoked at + 40 mV, the magnitude of EPSC at 150 ms post-stimulation was used to represent the NMDAR component, since the AMPAR component has returned to baseline at this time point (see Figure 1A). To examine exNMDAR currents, EPSCs mediated by sNMDARs were recorded while voltage clamping the neuron at + 40 mV in the presence of DNQX to block the AMPAR component and maintained at around 100 pA. After a > 90% blockade of sNMDAR currents by a 30-min-long application of MK801 (40 μM, EPSCs were evoked at 0.1 Hz during MK801 blockade), exNMDAR currents were induced by (i) the blockade of glutamate transporters by DL-threo-β-benzyloxyaspartic acid (TBOA, 20 μM; Milnerwood et al., 2010; Li S. et al., 2011), or (ii) a brief 10-pulse tetanus at 100 Hz as previously described (Luscher et al., 1998).

### Statistical Analysis

All data are presented as mean ± SEM. Statistical analyses were performed using JMP 13. Normality of data was examined using the Shapiro-Wilk test. Student’s *t*-tests were used for comparisons of normally distributed data between two groups. Two-way ANOVA was also used to compare the effect of stress on the input/output relationship of evoked fEPSPs. For data that are not normally distributed, Mann-Whitney tests were used for comparisons between two groups and Kruskal-Wallis tests and *post hoc* Wilcoxon tests were used for comparing data from three groups. Statistical significance (2-tailed) was set at *p* < 0.05.

## Results

### Acute Stress Enhances sNMDAR and Suppresses exNMDAR Functions

**FIGURE 2:**
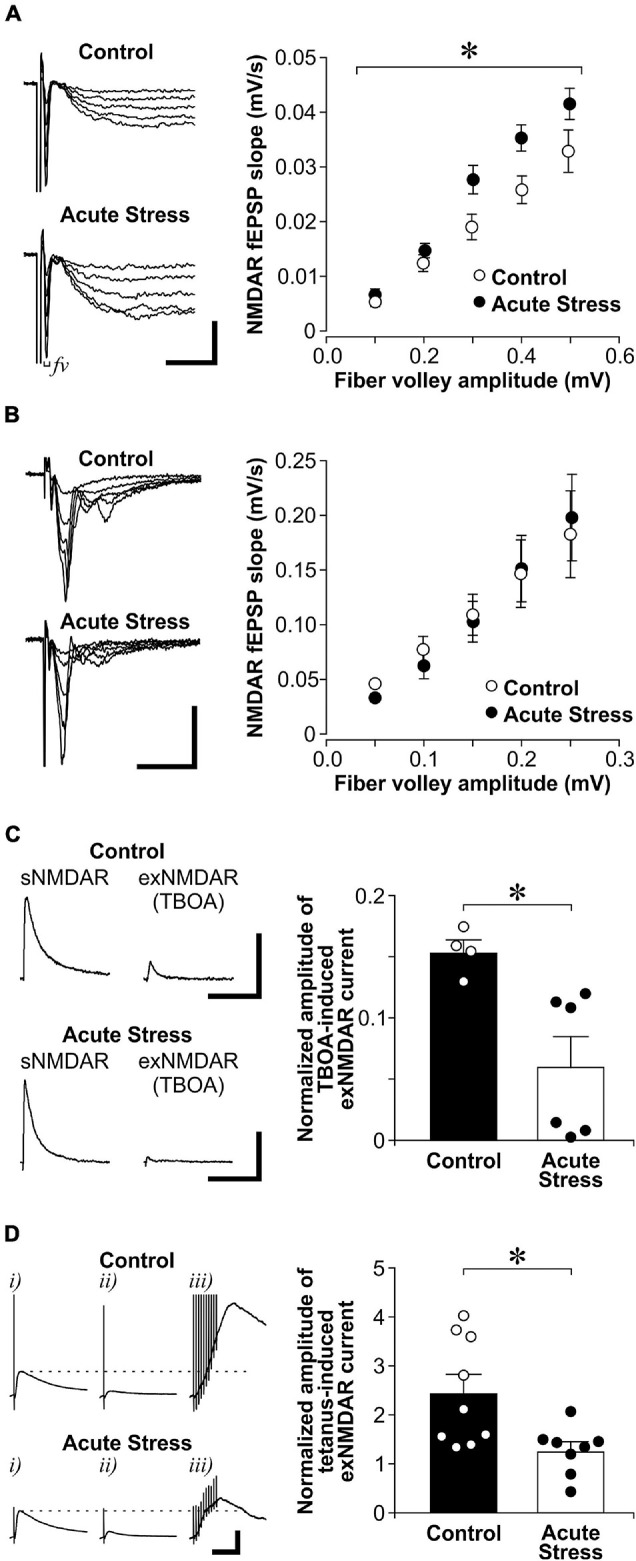
Acute stress enhances sNMDAR and reduces exNMDAR functions. **(A)** *Left*: Representative traces of evoked NMDAR-mediated evoked field excitatory postsynaptic potential (fEPSP) that were recorded from brain slices of control and acutely stressed rats (restrained for 30 min). Scale bar = 0.2 mV, 10 ms. *Right:* Scatter plots show the relationship between fiber volley (*fv*) amplitude and the slope of NMDAR fEPSP in control (6 recordings, 3 rats) and stressed rats (6 recordings, 3 rats). Mean ± SEM. *p < 0.05, interaction between the effect of stress and fiber volley amplitudes [repeated measures ANOVA, *F*(4, 40) = 3.46]. **(B)** *Left:* Representative traces of evoked AMPAR-mediated fEPSP that were recorded from brain slices of control and acutely stressed rats. Scale bar = 0.4 mV, 25 ms. *Right:* Scatter plots show the relationship between fiber volley amplitude and the slope of AMPAR fEPSP in control (15 recordings, 4 rats) and stressed rats (10 recordings, 3 rats). Mean ± SEM. **(C)** *Left:* Representative traces of sNMDAR and TBOA-induced exNMDAR currents that were recorded from brain slices of control and acutely stressed rats. Scale bar = 50 pA, 500 ms. *Right:* Histogram shows the normalized amplitude of exNMDAR currents recorded from slices of control (4 recordings, 4 rats) and acutely stressed rats (6 recordings, 3 rats). Mean ± SEM. *p < 0.05, Student’s *t*-test. **(D)** *Left:* Representative traces of evoked NMDAR mediated currents that were recorded from brain slices of control and acutely stressed rats before (*i*) and after a 30 min-long MK801 blockade (*ii*). Five minutes after washing out MK801, exNMDAR currents were induced by a brief tetanus stimulation [*iii*, 10-pulse (100 Hz)]. Dotted lines represent the level of sNMDAR current before MK801 blockade. Scale bar = 100 pA, 100 ms. *Right:* Histograms of normalized amplitude of exNMDAR currents recorded from brain slices of control (9 recordings, 9 rats) or acutely stressed rats (8 recordings, 5 rats). Mean ± SEM. *p < 0.05, Student’s *t*-test.labelF2

We first examined the effect of acute restraint stress on sNMDAR and exNMDAR functions in the hippocampal CA1 region of control and acutely stressed adult male rats. Acute restraint stress significantly increased CORT blood levels [CORT levels in controls: 48.5 ± 16.8 ng/ml; CORT levels in stressed rats: 495.4 ± 36.8 ng/ml; *t*(4) = 13.5; *p* = 1.73E-04]. In the presence of AMPAR and GABA_A_R antagonists, we isolated evoked fEPSPs that were mediated by sNMDARs (Figure 2A). To compare fEPSP between slices, we examined the relationship between the amplitude of fiber volley (*fv*; input), which represents the depolarization of synaptic inputs, and the slope of fEPSP (output). We found that acute stress significantly increases the input/output relationship of NMDAR-mediated fEPSPs when compared to controls {two-way ANOVA, stress—[*F*(1, 10) = 5.16, *p* = 0.046]; *fv* amplitudes (as repeated measures)—[*F*(4, 40) = 142.5, *p* = 4.24E-23]; stress × *fv* amplitudes—[*F*(4, 40) = 3.46, *p* = 0.016]}. However, acute stress did not affect the input/output relationship of AMPAR-mediated fEPSPs when compared to controls {Figure 2B; two-way ANOVA, stress—[*F*(1, 23) = 0.01, *p* = 0.918]; *fv* amplitudes (as repeated measures)—[*F*(4, 92) = 55.2, *p* = 1.21E-23]; stress × *fv* amplitudes—[*F*(4, 92) = 0.58, *p* = 0.681]}.

We next examined the impact of acute stress on exNMDAR function (Figure 2C). To isolate exNMDAR-mediated currents, we first blocked evoked sNMDAR currents by MK801. MK801 produced a sustained use-dependent blockade of NMDARs (Tse et al., 2019). We next induced a spillover of synaptic glutamate release by blocking glutamate transporter with TBOA to activate exNMDARs (Milnerwood et al., 2010; Li S. et al., 2011). Compared to controls, we found that acute stress reduces the normalized amplitude of exNMDAR currents [normalized by the amplitude of sNMDAR currents before MK801 blockade from the same cell; *t*(8) = 3.06, *p* = 0.016]. Apart from using TBOA, we used a brief tetanus (Luscher et al., 1998; 10-pulse at 100 Hz) to increase extracellular glutamate levels after MK801 blockade of sNMDAR to activate exNMDARs in another set of slices from stressed and control rats. Like the TBOA findings, we found that the amplitude of exNMDAR current in slices from acutely stressed rats is significantly smaller than that in slices from control rats (Figure 2D; U = 11, *p* = 0.018). These findings suggest that acute stress enhances sNMDAR and reduces exNMDAR function in dorsal hippocampal CA1 neurons.

### Acute Stress Reduces Ambient Glutamate-Mediated exNMDAR Currents

**FIGURE 3:**
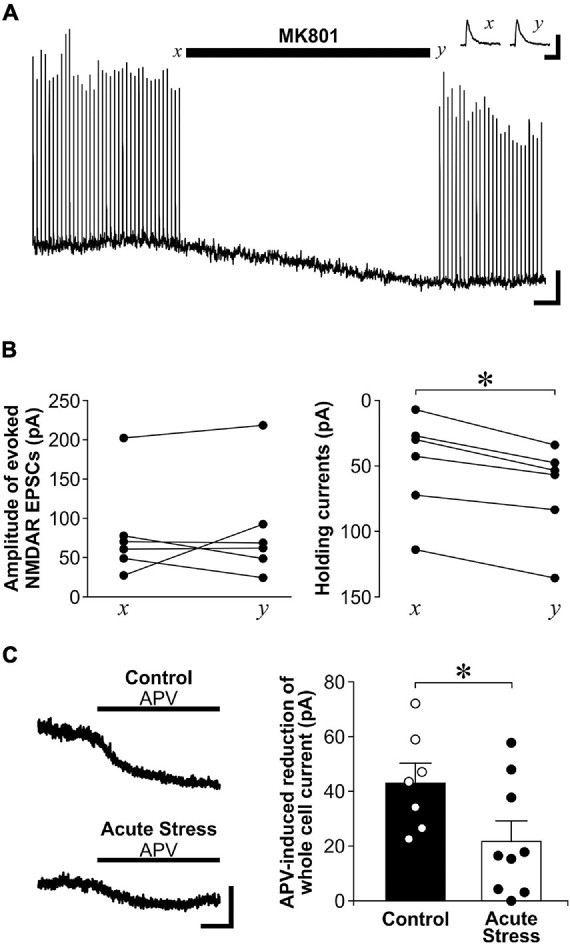
Acute stress reduces exNMDAR currents mediated by ambient glutamate. **(A)** Representative trace shows the effect of MK801 on evoked NMDAR-mediated EPSCs and whole-cell holding currents recorded from a CA1 neuron. Stimulations for evoking EPSCs were stopped at *x* and during MK801 application. MK801 reduced whole-cell holding currents. However, the amplitudes of evoked NMDAR-mediated EPSCs were not affected when stimulation was resumed at time *y*. The reduction of EPSC amplitude near the end of the trace was likely due to residual levels of MK801 in the recording chamber. Scale bar = 40 pA, 1 min. Inset: traces of sNMDAR before (*x*) and after (*y*) MK801 application. Scale bar = 200 pA, 200 ms. **(B)** Line plots show changes in evoked NMDAR-mediated EPSCs (*left*; 6 recordings, 3 rats) and whole-cell holding currents (*right*; 6 recordings, 3 rats) before (*x*) and after MK801 application (*y*). *p < 0.05, paired Student’s *t*-test; current at time *x* vs. time *y*. **(C)** *Left:* Representative traces of APV (50 μM) -induced blockade of whole-cell current of CA1 neurons in brain slices from control or acutely stressed rats. Scale bar = 40 pA, 1 min. *Right:* Histograms of the APV-induced blockade of whole-cell currents recorded from hippocampal slices of control (7 recordings, 3 rats) or acutely stressed rats (9 recordings, 4 rats). Mean ± SEM. *p < 0.05, Student’s *t*-test.

Since exNMDAR currents induced by TBOA and a brief tetanus after MK801 blockade was normalized by sNMDAR currents, the reduced exNMDAR function may also be due to the enhanced sNMDAR function caused by stress. To address this limitation, we used another method to directly measure exNMDAR function. exNMDARs can be activated by ambient levels of glutamate in brain slices, which can be revealed by the shift in whole-cell holding currents caused by NMDAR antagonists such as MK801 or APV (Le Meur et al., 2007; Papouin et al., 2012). To show that the shift in whole-cell currents caused by a NMDAR antagonist does not affect sNMDAR function, we recorded sNMDAR-mediated EPSCs from CA1 pyramidal neurons before and after perfusing slices with MK801. Although MK801 treatment caused a shift in whole-cell currents, it did not affect the amplitude of sNMDAR EPSCs (Figures 3A,B). Next, we compared the shift in whole-cell currents caused by APV between control and acutely stressed rats. Compared to slices from control rats, we found that APV-sensitive holding currents recorded from CA1 pyramidal neurons are significantly smaller in slices from acutely stressed rats [Figure 3C; *t*(14) = 2.17, *p* = 0.048]. These findings corroborated data from the spillover experiments to show that acute stress reduces exNMDAR function.

### Chronic Stress Suppresses exNMDAR Functions

**FIGURE 4:**
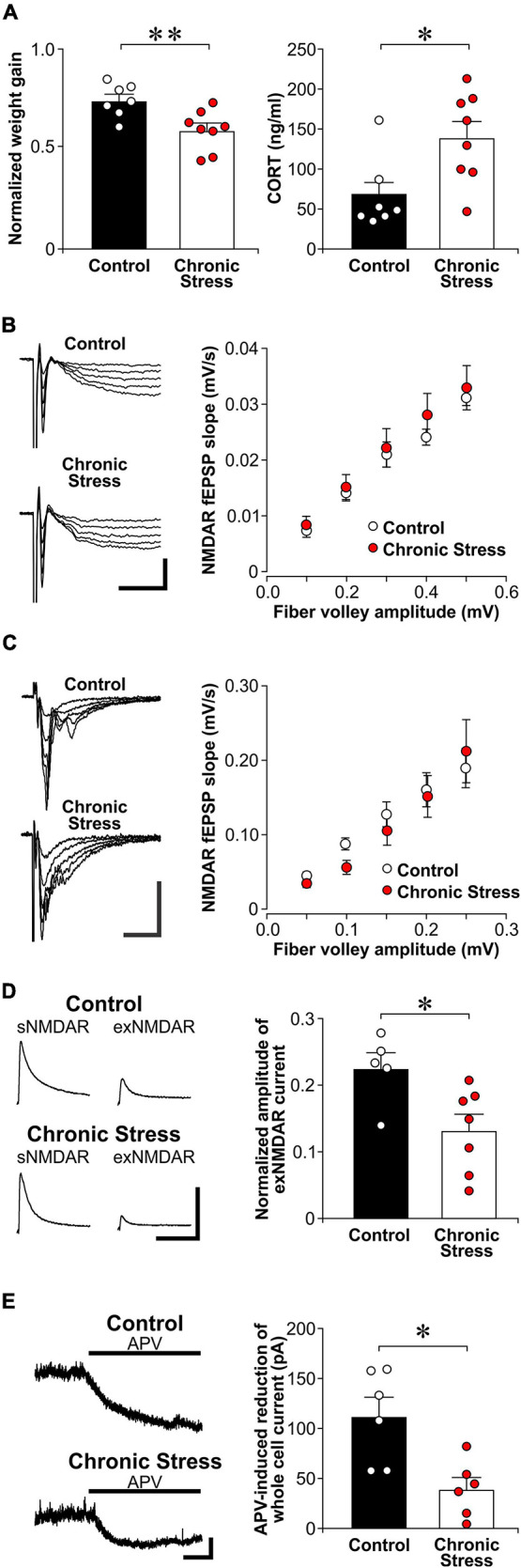
Chronic stress reduces exNMDAR functions. **(A)** *Left:* Histograms of normalized weight gain of control (*n* = 7) and chronically stressed rats (*n* = 8) in 21 days. *Right:* Histograms of CORT levels in trunk blood in control (*n* = 7) and chronically stressed rats (*n* = 8) collected right before brain slice preparation. Stressed rats were sacrificed 7 days within the end of chronic restraint stress. Mean ± SEM. *p < 0.05, **p < 0.01, Student’s *t*-test. **(B)** *Left:* Representative traces of evoked NMDAR-mediated field excitatory postsynaptic potential (fEPSP) that were recorded from brain slices of control and chronically stressed rats. Scale bar = 0.2 mV, 40 ms. *Right:* Scatter plots show the relationship between fiber volley amplitude and the slope of NMDAR fEPSP in control (7 recordings, 4 rats) and stressed rats (8 recordings, 4 rats). Mean ± SEM. **(C)** *Left:* Representative traces of evoked AMPAR-mediated fEPSP that were recorded from brain slices of control and chronically stressed rats. Scale bar = 0.4 mV, 25 ms. *Right:* Scatter plots show the relationship between fiber volley amplitude and the slope of AMPAR fEPSP in control (10 recordings, 4 rats) and stressed rats (13 recordings, 4 rats). Mean ± SEM. **(D)** *Left:* Representative traces of sNMDAR and TBOA-induced exNMDAR currents from brain slices of control or chronically stressed rats. Scale bar = 50 pA, 500 ms. *Right:* Histogram shows the normalized amplitude of exNMDAR currents recorded from brain slices of control (5 recordings, 4 rats) and chronically stressed rats (7 recordings, 6 rats). Mean ± SEM. *p < 0.05, Student’s *t*-test. **(E)** *Left:* Representative traces of APV (50 μM) -induced blockade of whole-cell current of CA1 neurons in brain slices from control and chronically stressed mice. Scale bar = 40 pA, 1 min. *Right:* Histograms of the APV-induced blockade of whole-cell currents recorded from control (6 recordings, 3 rats) and chronically stressed mice (6 recordings, 3 rats). Mean ± SEM. *p < 0.05, Student’s *t*-test.

After being stressed by restraint daily for 21 days, chronically stressed rats displayed lower body weight gain over 21 days of stress than control rats [Figure 4A; *t*(13) = 3.65, *p* = 0.003]. In addition, we found higher plasma CORT levels in chronically stressed rats when compared to non-stressed controls rats [*t*(13) = 2.74, *p* = 0.017]. Surprisingly, we found no difference in sNMDAR function between chronically stressed and control rats {Figure 4B; two-way ANOVA, stress—[*F*(1, 13) = 0.291, *p* = 0.599]; *fv* amplitudes (as repeated measures)—[*F*(4, 52) = 125.0, *p* = 5.24E-26]; stress × *fv* amplitudes—[*F*(4, 52) = 0.546, *p* = 0.703]}. Chronic stress also did not affect the input/output function of AMPAR-mediated fEPSP {Figure 4C; two-way ANOVA, stress—[*F*(1, 21) = 0.113, *p* = 0.741]; *fv* amplitudes (as repeated measures)—[*F*(4, 84) = 32.2, *p* = 2.94E-17]; stress × *fv* amplitudes—[*F*(4, 84) = 0.733, *p* = 0.572]}. Nonetheless, chronically stressed rats displayed lower TBOA-induced exNMDAR currents than control rats {Figure 4D, [*t*(10) = 2.69, *p* = 0.023]}. In addition, chronically stressed rats also showed lower APV-induced shifts in holding currents of CA1 neurons than control rats {Figure 4E, [*t*(8) = 3.31, *p* = 0.011]}. Taken together, chronic stress reduced exNMDAR function.

To further show the specific impact of acute stress on sNMDAR function, we performed whole-cell recordings of the NMDAR/AMPAR ratio of evoked EPSC. We found that acute stress significantly increased the NMDAR/AMPAR ratio of evoked EPSC when compared to control and chronic stress (Figure 1A; Kruskal-Wallis test, *Z* = 9.75, *p* = 0.008; *post hoc* Wilcoxon tests: control vs. acute stress: *p* = 0.010; acute stress vs. chronic stress: *p* = 0.012; control vs. chronic stress: *p* = 0.478).

### Acute and Chronic Stress Do Not Affect Presynaptic Glutamate Release

High presynaptic glutamate release might recruit exNMDARs during MK801 blockade and result in the reduced exNMDAR currents we observed in slices from stressed rats. If acute or chronic stress enhances presynaptic glutamate release, we expect MK801 would need a shorter time to block sNMDAR currents. However, comparing the time for blocking 50% of sNMDAR currents by MK801 between slices from control and acutely stressed rats (control: 10.2 ± 1.4 min; acute stress: 9.2 ± 1.0 min; [*t*(10) = 0.58; *p* = 0.578]), or between control and chronically stressed rats (control: 9.8 ± 0.7 min; chronic stress: 9.0 ± 1.1 min; [*t*(10) = 0.60; *p* = 0.562]), revealed no differences. We also compared the paired-pulse ratios of fEPSP at different interpulse intervals (25–200 ms) in slices from control and stressed rats. We found no differences in paired-pulse facilitation between the 3 groups {Figure 1B; two-way ANOVA, animal groups—[*F*(2, 34) = 0.370, *p* = 0.694]; interpulse interval (as repeated measures)—[*F*(3, 102) = 1.6, *p* = 0.187]; animal groups × interpulse interval—[*F*(6, 102) = 0.814, *p* = 0.561]}. Together, these findings suggest that the impact of stress on exNMDAR function is not related to changes in presynaptic glutamate release.

## Discussion

Stress-induced plasticity of NMDAR function depends on stress duration and the locations of these receptors. We show in this study that acute stress enhanced sNMDAR and reduced exNMDAR functions. Interestingly, reduced exNMDAR function was also induced by chronic stress. Apart from sNMDARs (Yuen et al., 2009; Tse et al., 2011), we reveal in this study that exNMDARs are highly susceptible to stress-induced regulation.

Our findings are in parallel to previous findings that while acute stress exposure increases sNMDAR function, this increase may be transient and may not be sustained by repeated exposure to the same stressor. In the prefrontal cortex (PFC), both acute restraint and forced swim stress have been shown to increase postsynaptic AMPAR and NMDAR responses (Yuen et al., 2009, 2012). However, recording these postsynaptic responses from rats that were stressed by a similar restraint stressor daily revealed a decrease in synaptic AMPAR and NMDAR function after 7 days when compared to controls (Yuen et al., 2012). In the same study, 7-day restraint stress did not affect AMPAR function in the CA1. Finally, both synaptic AMPAR and NMDAR responses recorded from granule cells of the dentate gyrus were not affected in rats that have received 21 daily episodes of chronic unpredictable stress (Karst and Joels, 2003). Since there was a mean delay of 3 days between the end of chronic stress and electrophysiology recording, we cannot rule out the possibility that sNMDAR was enhanced immediately after chronic stress but returned to baseline when we sacrificed the animals. Even if sNMDAR function is transiently increased after chronic stress, this change is unlike the persistent decrease in exNMDAR function.

We found that exNMDAR function was reduced by both acute and chronic restraint stress. Although glutamate spillover caused by either a brief tetanus or the blockade of glutamate transporters has been used to estimate exNMDAR function, these indirect methods have limitations. To compare exNMDAR function between slices, exNMDAR function was normalized by sNMDAR currents from the same slice in the current study. The increase in sNMDAR function caused by acute stress could be a factor that may lead to a decrease in normalized exNMDAR function. The more direct method of estimating exNMDAR function by using the blockade of exNMDAR-mediated whole-cell currents was used to address this limitation (Le Meur et al., 2007; Yao et al., 2018). The spillover method may also target a specific population of exNMDARs. In Figure 3, we found that the MK801 treatment we used to block sNMDARs could block exNMDARs that are activated by ambient glutamate. This finding suggests that the spillover approach recruits a different population of exNMDARs (also called perisynaptic NMDARs) (Mitani and Komiyama, 2018) that are not sensitive to ambient glutamate. Despite these limitations, we found that both acute and chronic stress reduce exNMDAR currents that are sensitive to ambient glutamate. In addition, chronic stress reduced TBOA- and tetanus-induced exNMDAR currents without affecting sNMDAR function. Together, our findings suggest that exNMDARs are reduced by both acute and chronic stress.

Mechanisms underlying the reduction of exNMDAR function after stress remains to be determined. After acute stress, the reduction of exNMDARs may be due to surface trafficking of these receptors to synapses for enhancing sNMDAR function (e.g., see Tovar and Westbrook, 2002; Groc et al., 2006). Nonetheless, how a reduction of exNMDARs was maintained when sNMDAR function is no longer enhanced after chronic stress is not clear. Since the expression of glutamate transporter can be reduced by chronic stress (Liu et al., 2016, 2017; Zhu et al., 2017), the increase in ambient glutamate after a reduction in transporter function may downregulate exNMDAR expression, which are more sensitive to ambient glutamate than sNMDARs. The loss of astrocytes, a suggested source of glutamate for activating exNMDARs (Carmignoto and Fellin, 2006), in the stressed brain (Czeh et al., 2006) could also contribute to reduced exNMDAR function.

A loss of exNMDAR function could contribute to the impact of chronic stress on hippocampal function. Since exNMDARs mediate NMDAR spikes (Suzuki et al., 2008; Oikonomou et al., 2012) that amplify synaptic signals (Harris and Pettit, 2008) and enhance neuronal excitability (Sah et al., 1989; Oikonomou et al., 2012), reduced exNMDAR function could contribute to the negative impact of chronic stress on rats’ performance in spatial working and reference memory tasks (Luine et al., 1994; Henningsen et al., 2009; Veena et al., 2009). However, not all hippocampus-related cognitive functions are impaired by chronic stress. For instance, contextual fear conditioning is enhanced in chronically stressed rats (Conrad et al., 1999; Sandi et al., 2001). Given that exNMDARs are associated with forgetting mechanisms such as long-term depression (Liu et al., 2013), reduction in exNMDARs may be related to the enhanced encoding of aversive information in stressed mice. Recently, we have shown that mice that are susceptible to a chronic social defeat stressor show enhanced negative memory engrams formation in the hippocampal CA1 region (Zhang et al., 2019). In addition, we observed a reduction of exNMDAR function in this hippocampal region of mice that are susceptible, but not resilient to this stressor (Tse et al., 2019). These findings suggest that the enhanced encoding of aversive information could be related to a reduction in exNMDARs. Further studies could investigate if enhancing exNMDAR function, for example using drug-coating nanoparticles that are too big to enter synaptic cleft (Savchenko et al., 2016; Tse et al., 2019; Valente et al., 2020), will ameliorate the enhancement of aversive memory in stressed rats.

## Conclusion

In conclusion, we show that apart from sNMDAR function, exNMDAR function can be modulated by both acute and chronic stress. Given the emerging functional roles of exNMDARs, changes in exNMDAR function could be related to the pathogenesis of stress-related brain disorders.

## Data Availability Statement

The raw data supporting the conclusions of this article will be made available by the authors, without undue reservation.

## Ethics Statement

The animal study was reviewed and approved by Facility Animal Care Committee, McGill University.

## Author Contributions

YT, MN, and TW conducted all experiments. YT and TW designed the study. YT, AL, and TW analyzed the data and wrote the manuscript. All authors have approved the submitted version.

## Conflict of Interest

The authors declare that the research was conducted in the absence of any commercial or financial relationships that could be construed as a potential conflict of interest.

## Publisher’s Note

All claims expressed in this article are solely those of the authors and do not necessarily represent those of their affiliated organizations, or those of the publisher, the editors and the reviewers. Any product that may be evaluated in this article, or claim that may be made by its manufacturer, is not guaranteed or endorsed by the publisher.
